# Antenatal depression and the association of intimate partner violence among a culturally diverse population in southeastern Norway: A cross-sectional study

**DOI:** 10.18332/ejm/150009

**Published:** 2022-07-18

**Authors:** Thea Cathrine Melby, Nina Benedicte Sørensen, Lena Henriksen, Mirjam Lukasse, Eva Marie E. Flaathen

**Affiliations:** 1 Department of Nursing and Health Promotion, Faculty of Health Sciences, Oslo Metropolitan University, Oslo, Norway; 2 Division of General Gynaecology and Obstetrics, Oslo University Hospital, Oslo, Norway; 3 Department of Nursing and Health Sciences, Faculty of Health and Social Sciences, University of South-Eastern Norway, Kongsberg, Norway

**Keywords:** antenatal depression, depression during pregnancy, immigrant women, physical intimate partner violence, sexual intimate partner violence, emotional intimate partner violence

## Abstract

**INTRODUCTION:**

Antenatal depression and intimate partner violence (IPV) are independently associated with adverse short- and long-term health effects for women and their children. The main aim of the study was to investigate the prevalence of antenatal depression and the association between symptoms of antenatal depression and physical, emotional and sexual abuse in a culturally diverse population attending antenatal care.

**METHODS:**

A cross-sectional study was conducted with 1812 culturally diverse pregnant women from Safe Pregnancy, a randomized controlled trial to test the effect of an intimate partner violence intervention for abused women in southeastern Norway.

**RESULTS:**

More than one in ten women (14%) reported symptoms of antenatal depression. Women with symptoms of antenatal depression were significantly younger and single, had lower educational level, more limited economic resources and were more likely to use tobacco and to report negative experiences regarding alcohol consumption, including that of her partner, compared to women with no symptoms of depression. A total of 15.4% of the women reported experiences of some form of IPV during their lifetime. Most women reported previous experiences of IPV rather than recent experiences. Women with a history of IPV were significantly more likely to report symptoms of antenatal depression, after adjusting for confounding factors (AOR=1.96; 95% CI: 1.35–2.83).

**CONCLUSIONS:**

Women who reported symptoms of antenatal depression were significantly more likely to have experienced physical, emotional and sexual IPV than women with no history of IPV. It is important to identify women at risk of antenatal depression in order to offer appropriate services during pregnancy.

## INTRODUCTION

Pregnancy and the transition to parenthood involve many physiological and psychosocial changes and is recognized as a vulnerable period that can increase the risk of onset or relapse of mental illness^1^.

Antenatal depression is the most common psychiatric disorder/mental illness during pregnancy^1^. Symptoms, such as feelings of sadness, emptiness, guilt, loss of interest, fatigue, loss of appetite, energy and sleep changes, can occur at any time during the pregnancy and range from mild to severe^1^. There is a tendency to focus on physical maternal and fetal health during pregnancy, rather than mental health^1^. Thus, depression during pregnancy is often undetected by health professionals and untreated^1^. Nevertheless, depression during the pregnancy is common^1-5^.

The prevalence of antenatal depression in high-income countries varies between 7% and 20% whereas rates of 20% or more have been reported in low- and middle-income countries^1^. The range in prevalence of antenatal depression in different studies depends on the setting, study design, measurements, and definitions^1^. In a Norwegian prospective cohort study, 13% of 749 ethnically diverse women reported depressive symptoms during pregnancy^3^. The prevalence of antenatal depression was significantly higher among minority women from Middle Eastern countries and South Asia compared to women originating from Norway and Western Europe^3^. In 2019, one in three women who gave birth in southeastern Norway, were not born in Norway^6^.

There is increasing evidence that antenatal depression can be a predictor for negative birth outcomes, including low birth weight and prematurity^7^. Further, antenatal depression is associated with short- and long-term adverse health effects for both mother and child, including substance abuse, inadequate prenatal care, post-traumatic stress, post-partum depression and impaired child behavioral, cognitive and emotional development^1,7^.

Identification of risk factors for antenatal depression is crucial. In a systematic review, Biaggi et al.^1^, identified a range of psychosocial, medical and demographic risk factors for antenatal depression such as a history of depression, substance abuse, marital difficulties, lack of a partner or social support, poverty, ethnicity, unintended pregnancy and intimate partner violence. Lack of extended family and social support may be an important stress factor and indicator for sustained or recurrent depressive symptoms for immigrant pregnant women^5,8^. However, results from a systematic review and meta-analysis conducted in high-income countries investigating risk of antenatal depression in the perinatal period among migrant women, are inconclusive^8^.

Intimate partner violence during pregnancy is prevalent globally and a public health concern^9,10^. The term ‘intimate partner violence’ includes physical aggression, sexual coercion, psychological abuse, or controlling behavior perpetrated by a current or former partner^10^. Although the estimated prevalence of IPV during pregnancy varies worldwide, most studies report a prevalence of physical and sexual IPV of 4–9%^11^. In a meta-analytic review, James et al.^9^ found a prevalence of physical, emotional and sexual IPV during pregnancy of 13.2% in developed countries. Norwegian studies have found a prevalence of IPV during pregnancy ranging 1–5%^12,13^. Previous Norwegian studies included few women from minority populations, thus a knowledge gap regarding IPV within different immigrant groups and cultural setting exists^12,13^.

IPV is an important direct or indirect cause of women’s adverse physical, mental and reproductive health^14^. IPV during pregnancy is associated with adverse pregnancy outcomes for both the mother and her infant including miscarriage, preterm birth, small for gestational age babies, stillbirth and negatively affects bonding between women and their babies^14,15^. IPV affects women in all social settings and among all socioeconomic, religious, and cultural groups^10^. Nevertheless, demographic, behavioral, and social risk factors such as abuse before pregnancy, low educational level, being single, unintended or unwanted pregnancy, being of low socioeconomic status, or having a partner who abuses alcohol are identified as significant risk factors for experiencing IPV during pregnancy^9^. Women with an immigrant background have been identified as particularly vulnerable to IPV, as they are likely to be overrepresented in groups that have limited economic resources and low educational level^16^. Factors related to their migration context, such as language barriers, family separations, social isolation, cultural differences and flight from war and violence may increase their vulnerability for IPV exposure^16^. Patriarchal regimes and unequal juridical and financial rights for women also increase the risk of IPV exposure^17^.

Although previous studies offer evidence of the independent negative health consequences for women experiencing antenatal depression^3,7,18,19^ or IPV^14,15,20^, only few European, Nordic and Norwegian studies have investigated the association between antenatal depression and IPV in an unselected culturally diverse population^1,18,21^. Further, most of the studies only include physical and/or sexual IPV^1,18^. The main aim of this study was to investigate the prevalence of antenatal depression and the association between antenatal depression and physical, emotional and sexual IPV in a culturally diverse population attending antenatal care in southeastern Norway.

## METHODS

### Study design

This cross-sectional study included baseline data from the Safe Pregnancy randomized controlled trial (RCT) aiming to test the effect of a culturally sensitive intervention to promote quality of life, use of safety behaviors and prevent intimate partner violence (IPV) among Norwegian, Pakistani and Somali pregnant women^22^. The rationale for including women with Pakistani and Somali backgrounds was that they are among the largest non-western immigrant groups in Norway with high fertility rates^23^, and have patriarchal cultural norms that may permit IPV^17^.

### Setting, sample and procedure

**Figure 1:**
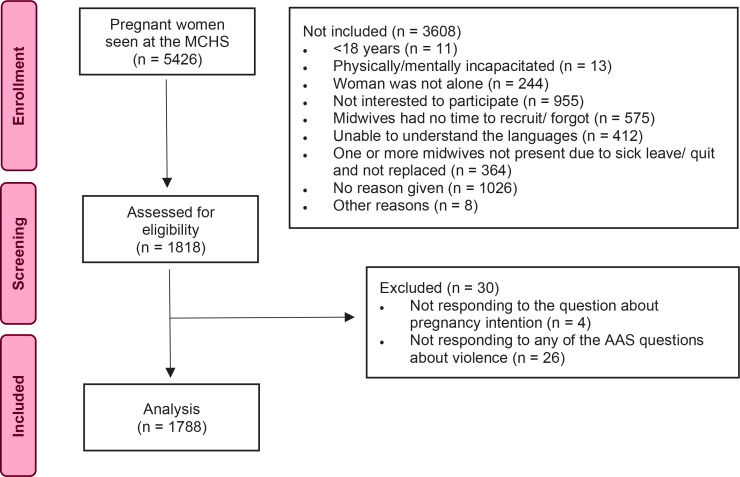
Participant selection flow chart

The Safe Pregnancy study was conducted in a routine antenatal care setting at 19 maternal and child health centers (MCHC) between January 2018 and July 2019^22^. A total of 1818 of 5426 pregnant women participated in the study (Figure 1). The most common reasons for women not to participate were due to lack of interest or that the midwives did not have time or forgot to recruit the women. In the current study, six women were excluded due to lack of information regarding depressive symptoms. The study sample consisted of 1812 women.

Midwives recruited women at any gestational age throughout the pregnancy. The inclusion criteria were pregnant women aged ≥18 years who were meeting alone with the midwife^22^. Women who did not understand Norwegian, English, Urdu or Somali or did not have the mental or physical capacity to answer the questionnaire were excluded^22^. The participating women answered the baseline questionnaire using a tablet. The tablet-based questionnaire (Q1) included questions about sociodemographic and socioeconomic backgrounds and obstetric history. Validated instruments measured quality of life, physical and mental health, depressive symptoms and IPV^22^. All study material were translated by professionals into Norwegian, English, Urdu and Somali.

### Variables

*The Edinburgh Postnatal Depression Scale*

The main outcome variable, symptoms of antenatal depression, was measured using the short-matrix version of the Edinburgh Postnatal Depression Scale (EPDS)^24^. The construct of the short version of the EPDS included women of reproductive age^24^. The instrument consists of five items that showed the highest correlation to the full version of the EPDS (r=0.96) and shows good psychometric properties and may replace the full version in questionnaire studies^24^. The instrument asks five questions about how you have felt in the past seven days: 1) Have you felt sad or miserable?, 2) I have been anxious or worried for no good reasons, 3) I have felt so unhappy that I have had difficulties sleeping, 4) I have blamed myself unnecessarily when things went wrong; and 5) I have looked forward with enjoyment to things. The answer-options were ‘no, never’, ‘not very often’, ‘yes, sometimes’, and ‘yes, most of the time’. The maximum score is 15 and each item ranged from 0 (absence of symptoms) to 3 (maximum severity of symptoms)^24^. In this study, a total score of >7 indicates symptoms of antenatal depression. The score is comparable with the full-scale version of the EPDS where >10 indicates a moderate level of depressive symptoms^24^. This cutoff resulted in two categories. Women with moderate to severe depressive symptoms with a score >7 were categorized as ‘symptoms of antenatal depression’ whereas women with milder or no depressive symptoms with a score <6 were categorized as ‘no symptoms of depression’. The EPDS score is an indicator of depressive symptoms rather than the clinical diagnosis of depression^24^.

*The Abuse Assessment Screen*

The exposure variables were measured by a modified version of the validated Abuse Assessment Screen (AAS)^25,26^. The AAS has been validated in an obstetric and gynecological outpatient population and has shown good sensitivity (94%) and fair to good specificity (55-99%)^25,26^. The AAS consisted of the following five descriptive questions measuring fear of partner and emotional, physical and sexual IPV: 1) Have you ever been afraid of your partner or someone else?; 2) Have you ever experienced that a partner or ex-partner has done things to make you feel afraid of them?; 3) Done things to try to intimidate you or to control your thoughts, feelings, or actions?; 4) Hit, kicked, pulled you by your hair or otherwise physically hurt you?; and 5) Forced you to have sexual activities against your will?. The first question overlapped with the second question and were excluded from the analysis. Question 2 to 5 were categorized as ‘fear’, ‘emotional IPV’, ‘physical IPV’ and ‘sexual IPV’, respectively^22^. The answer options were ‘never’, ‘yes, previously’, ‘yes, during the past 12 months before this pregnancy’ and ‘yes, since the start of the pregnancy’^22^. The responses were classified as ‘no IPV’, ‘previous IPV’, ‘recent IPV’ and ‘previous and recent IPV’^22^. Experiences of IPV were determined by a positive answer to at least one of the four questions. Women who reported experiences of fear, emotional, physical and/or sexual IPV were considered to have experienced IPV and categorized as ‘any lifetime IPV’.

*Background variables*

Background characteristics of the women were derived from Q1. Women reported sociodemographic, socioeconomic and obstetric status by selecting predefined categories. Women reported age in years and the variable was recoded into four categories (Table 1). The women were asked about their civil status and the answer options were ‘married or living with partner’, ‘single’ and ‘other’. Civil status was dichotomized and coded as ‘married or living with partner’ or ‘other’ (Table 1). The question about education was coded in years. It included five categories that were merged into three categories; the category ‘≤13 years’ included women who had no education, primary school and high school education (Table 1). Occupation status was recoded from seven categories into ‘employed’ or ‘not employed’. The women were asked about their joint family income last year and six categories were merged and recoded into four categories (Table 1). Women were asked whether their mother tongue was Norwegian, Somali, Urdu, English or other and dichotomized into Norwegian and other (Table 1). Using mother tongue as a determining factor can be a true indicator of understanding and orienting oneself in a different culture^27^. Pregnancy intention was a dichotomous variable including the answer options ‘yes’ or ‘no’ (Table 1). Questions about tobacco use and snuff were merged and labeled as ‘tobacco use’ (Table 1). The questions about alcohol addressed negative experiences regarding problematic behavior related to alcohol consumption during the last year, including those of the woman’s partner (Table 1).

**Table 1:** Participant characteristics by symptoms of antenatal depression among women in the Safe Pregnancy study, Norway 2018–2020 (N=1812)

Characteristics	*Symptoms of antenatal depression (n=254)*	*No symptoms of antenatal depression (n=1558)*	p*
	*n (%)*	*n (%)*	
**Age** (years)			0.46
<25	15 (5.9)	80 (5.2)	
25–30	89 (35.2)	615 (39.8)	
31–35	104 (41.1)	567 (36.7)	
>35	45 (17.8)	283 (18.3)	
Missing	14		
**Civil status**			<0.001
Married/living with partner	222 (91.4)	1472 (96.8)	
Other	21 (8.6)	48 (3.2)	
Missing	49		
**Education level** (years)			0.15
High school ≤13 years	76 (30)	393 (25.3)	
College/university less than 4 years	71 (28.1)	519 (33.4)	
College/university more than 4 years	106 (41.9)	643 (41.4)	
Missing	4		
**Occupation**			0.15
Employed or self-employed	198 (78.6)	1281 (82.4)	
Not employed	54 (21.4)	274 (17.6)	
Missing	5		
**Joint family income last year** (NOK)			<0.001
>599000	61 (24.3)	232 (14.9)	
600000–999000	104 (41.4)	710 (45.7)	
>1000000	55 (21.9)	475 (30.6)	
Do not know	31 (12.4)	135 (8.7)	
Missing	9		
**Mother tongue**			0.09
Norwegian	175 (69.7)	1150 (74.8)	
Other	76 (30.3)	387 (25.2)	
Missing	24		
**Parity**			0.87
0	120 (47.4)	743 (48)	
≥1	133 (52.6)	806 (52)	
Missing	10		
**Pregnancy planned**			0.001
Yes	190 (75.1)	1304 (83.9)	
No	63 (24.9)	251 (16.1)	
Missing	4		
**Tobacco**			0.001
Yes	18 (7.1)	44 (2.8)	
No	236 (92.9)	1506 (97.2)	
Missing	8		
**Alcohol**			<0.001
Yes	38 (16.3)	85 (5.7)	
No	195 (83.7)	1410 (94.3)	
Missing	84		
**Alcohol partner**			<0.001
Yes	34 (14.7)	55 (3.7)	
No	198 (85.3)	1420 (96.3)	
Missing	105		

* Chi-squared test. NOK: 1000 Norwegian Krone about 101 US$.

### Statistical analysis

Descriptive data are presented as frequencies (n) and proportions (%) for categorical variables. Cross-tabulations and Pearson’s chi-squared tests were used to determine percentages and compare the prevalence of symptoms of antenatal depression based on categorical variables, such as sociodemographic, socioeconomic and obstetric factors, and a history of IPV. To examine the association between symptoms of antenatal depression and a history of IPV, binary logistic regression analyses were performed to calculate the crude odds ratio (OR) and adjusted odds ratio (AOR) with a 95% confidence interval (CI). Covariates with a p<0.1 in the univariate analyses and age were included in the multivariate analyses. A p<0.05 was considered as statistically significant. All tests were two-sided, and the analyses were performed using IBM SPSS Statistics^27^ (IBM Corp., Armonk, NY, USA).

### Ethical considerations

The Regional Committee for Medical and Health Research Ethics (REC) approved the Safe Pregnancy study. The study followed the WHO guidelines for researching violence against women^28^ and the Helsinki Declaration of research ethics^29^. The women received verbal and written information about the study and midwives received signed consent forms from all the participants. Data were anonymized before analysis. All women, irrespective of experiencing IPV, received and appointment card featuring a list of phone numbers and websites to community resources^22^.

## RESULTS

The women’s mean age was 31.3 years. Most women were married or living with their partners, were employed and had higher educational level. A total of 25.5% of the participating women were non-native Norwegian speakers (Pakistani 4.1%, Somali 0.9%, English 0.8% and others 19.7% [data not provided in tables]). There were no significant differences between native Norwegian speakers and non-native Norwegian speakers regarding reported symptoms of antenatal depression (p=0.09). Further, single women and women with limited economic resources were significantly more likely to report symptoms of antenatal depression.

More than one in ten (14%) women reported symptoms of antenatal depression (Table 1). A total of 275 (15.4%) of the women reported to have experienced some form of IPV during their lifetime (Table 2). The most common form of violence was emotional IPV (12.2%), and fear of partner (10.6%) and women reported previous rather than recent experiences of IPV. Women who had experienced any lifetime IPV, fear, emotional, physical and sexual IPV were significantly more likely to report symptoms of antenatal depression compared with women with no history of IPV.

**Table 2:** Symptoms of antenatal depression by a history of intimate partner violence (IPV) among women in the Safe Pregnancy study, Norway 2018–2020 (N=1812)

*The AAS*	*Total(n=1812) n (%)*	*Symptoms of antenatal depression (n=254) n (%)*	*No symptoms of antenatal depression (n=1558) n (%)*	p*
**Any lifetime IPV**				<0.001
No	1512 (84.6)	176 (70.7)	1336 (86.9)	
Yes	275 (15.4)	73 (29.3)	202 (13.1)	
Missing	25			
**Fear**				<0.001
No	1598 (89.4)	193 (77.5)	1405 (91.3)	
Previous	176 (9.8)	45 (18.1)	131 (8.5)	
Recent	12 (0.7)	9 (3.6)	3 (0.2)	
Previous and recent	2 (0.1)	2 (0.8)	0 (0)	
Missing	24			
**Emotional IPV**				<0.001
No	1570 (87,8)	193 (77.2)	1377 (89.5)	
Previous	200 (11.2)	46 (18.4)	154 (10.0)	
Recent	11 (0.6)	6 (2.4)	5 (0.3)	
Previous and recent	7 (0.4)	5 (2)	2 (0.1)	
Missing	24			
**Physical IPV**				<0.001
No	1694 (94.7)	218 (87.2)	1476 (95.9)	
Previous	87 (4.9)	25 (10)	62 (4.0)	
Recent	4 (0.2)	4 (1.6)	0 (0)	
Previous and recent	4 (0.2)	3 (1.2)	1 (0.1)	
Missing	23			
**Sexual IPV**				<0.001
No	1731 (96.8)	231 (92.8)	1500 (97.5)	
Previous	56 (3.1)	17 (6.8)	39 (2.5)	
Recent	1 (0.1)	1 (0.4)	0 (0)	
Missing	24			

* Chi-squared test.

Further, women who reported symptoms of antenatal depression were significantly more likely to have an unintended pregnancy (24.9% vs 16.1%), to smoke (7.1% vs 2.8%) and to have experienced negative behavior related to her own (16.3% vs 5.7%) and her partners (14.7% vs 3.7%) alcohol consumption during the past 12 months than women with no symptoms of depression.

In the univariate logistic regression analysis, experiences of any lifetime IPV and all the different forms of IPV were significantly associated with symptoms of antenatal depression (Table 3). The associations between symptoms of antenatal depression and experiences of IPV were attenuated but remained statistically significant in the adjusted analysis when controlling for age, civil status, joint family income, mother tongue, tobacco use and negative behavior in relation to alcohol consumption for both the woman and her partner. Women who had experienced any IPV during their lifetime had two times higher odds of reporting symptoms of antenatal depression (AOR=1.96; 95% CI: 1.35–2.83) compared with women with no history of IPV. Similar results were found regarding fear of partner (AOR=2.02; 95% CI: 1.32–3.07), emotional IPV (AOR=1.83; 95% CI: 1.23–2.74), physical IPV (AOR=2.35; 95% CI: 1.37– 4.04) and sexual IPV (AOR=2.09; 95% CI: 1.03–4.23).

**Table 3:** Crude and adjusted odds ratios for symptoms of antenatal depression by a history of intimate partner violence (IPV) among women in the Safe Pregnancy study, Norway 2018–2020 (N=1812)

*The AAS*	*Total*	*Symptoms of antenatal depression*			
	*n (%)*	*OR*	*(95% CI)*	*AOR*	*(95% CI)*
No IPV (Ref.)	1512 (84.6)	1		1	
Any IPV	275 (15.4)	2.74	(2.01–2.74)	1.96	(1.35–2.83)
Fear	190 (10.6)	3.04	(2.15–4.30)	2.02	(1.32–3.07)
Emotional IPV	218 (12.2)	2.53	(1.80–3.54)	1.83	(1.23–2.74)
Physical IPV	95 (5.3)	3.44	(2.20–5.39)	2.35	(1.37–4.04)
Sexual IPV	57 (3.2)	3.0	(1.87–5.33)	2.09	(1.03–4.23)

AOR: adjusted odds ratio. Adjusted for background variables: age, civil status, joint family income, mother tongue, unintended pregnancy, tobacco use, alcohol, and alcohol partner.

## DISCUSSION

The main finding in this study is that more than one in ten women (14%) attending routine antenatal care in southeastern Norway, reported symptoms of antenatal depression measured with the short-matrix EPDS^24^. Approximately one in four women were non-native Norwegian speakers. There was no significant difference in reported symptoms of antenatal depression between native Norwegian speakers and non-native Norwegian speakers. Further, 15.4% of the women reported to have a history of physical, emotional and/or sexual IPV during their lives. Women with a history of fear of a partner, physical, emotional and/or sexual IPV were significantly more likely to report symptoms of antenatal depression compared to women with no history of IPV.

The prevalence rate of women reporting symptoms of antenatal depression in our study is comparable with the results from previous studies^1-3,5^. In a systematic review, the prevalence of antenatal depression was 15% in a sample of 28248 pregnant women^2^. Similar findings were reported in a longitudinal study including a Swedish national sample of 1558 pregnant women in gestational week 35–36^4^ and in a Norwegian prospective cohort study^3^ with a prevalence of 17% and 13%, respectively. Both studies used the EPDS with a cut-off score of ≥10 indicating moderate symptoms of antenatal depression, which is comparable to the short-matrix version of the EPDS with a cut-off score of ≥7 that we used in our study^3,4,24^. In another longitudinal study including a national sample of 2926 Swedish women, Rubertsson et al.^5^, found a prevalence of antenatal depression of 13.7% in early pregnancy. Symptoms of depression are prevalent in any period during the pregnancy^2,3,5^. It is crucial to identify women at risk to reduce the prevalence of depression during pregnancy and thus reduce the adverse short- and long-term health effects for women, their babies and families.

Non-native Norwegian speaking women in our study did not have a significant risk of developing symptoms of antenatal depression compared to native Norwegian speakers. Results from a systematic review and meta-analysis, conducted in high-income countries investigating risk of antenatal depression in the perinatal period among migrant women, are inconclusive^8^. Anderson et al.^8^ showed that studies from Canada found an increased risk of antenatal depression associated with migrant status, whereas studies from USA and Australia found no association between antenatal depression and migrant status. Additionally, studies from USA found a decreased risk of antenatal depression associated with migrant status^8^. Compared to our study, a Norwegian population-based prospective cohort found that Middle Eastern (19.5%) and South Asian (17.5%) women had significantly higher risk for symptoms of antenatal depression compared to Western European women (8.6%)^3^. A Swedish longitudinal study of a national sample of pregnant women found that native language other than Swedish were associated with antenatal depression^5^. Few women with Pakistani and Somali background participated in our study. Studies have showed that migrant women may be sceptic to participate in research due to cultural differences, language barriers and lack of trust regarding anonymity and confidentiality^30-32^. Migrants from Pakistan, Somalia and Central and Eastern Europe constitute the vast majority of childbearing women in Norway^23^. Thus, it is plausible that non-native Norwegian women in our study originated from countries in Central and Eastern Europe, which may be associated with lower odds of experiencing antenatal depression^5^. The literature about symptoms of depression in pregnancy among populations of ethnic minorities is limited^3,5,8^. There is a need of awareness in health policy about the importance of adapting adequate services for these groups to meet their specific needs for care during pregnancy.

The present study demonstrates a significant association between antenatal depression and physical, emotional and sexual IPV separately. Compared to our study, most studies report prevalence estimates and odds ratio for having experienced ‘any violence’ including physical, emotional and sexual violence^19^. A systematic review conducted in high-, middle-, and low-income countries investigating risk factors associated with antenatal depression concluded that IPV was one of the strongest predictors for developing antenatal depression^1^. These findings are concurrent with findings in another systematic review and meta-analysis that analyzed the association between antenatal depression and any IPV^19^. Pooled estimates from cross-sectional studies in the current systematic review, showed that women with antenatal depression had a 3- to 5-fold increased odds of having experienced any IPV during their lifetime, during the past year and during pregnancy^19^. According to Howard et al.^19^, there is a need of high-quality evidence on how maternity and mental health services should address IPV and improve health outcomes for women in the antenatal period.

Few women in our study reported experiences of IPV during the past year or during the pregnancy. Although our results suggest an association between antenatal depression and recent experiences of IPV, the results should be interpreted with caution. A previous review found a strong association between antenatal depression and women with a history of both previous and recent history of IPV^18^. Women with a history of IPV are more prone to depression in general and depression is known to be an important risk factor for developing antenatal depression^1^. Thus, it is plausible that women who have endured IPV earlier in life also may have been depressed prior to becoming pregnant. Howard et al.^19^, found that women suffering from antenatal depression had an increased risk of experiencing IPV during the first year after giving birth. These findings suggest that a two-way association between experiences of IPV and antenatal depression is likely, in which symptoms of depression may increase women’s vulnerability to IPV and having experienced IPV can increase the risk of depression during pregnancy^19^.

### Strengths and limitations

A major strength of the study was the large sample of culturally diverse women attending antenatal care in southeastern Norway. Another strength of this study was the inclusion of validated, standardized and commonly used instruments measuring antenatal depression and IPV in the obstetric population^24-26^. The study provided a broad picture of the violence by including fear of a partner, physical, emotional and sexual violence separately. Additionally, the AAS included recent and previous experiences of IPV.

A cross-sectional study can identify and describe possible associations between selected variables but does not provide information about causal relationships^33^. In our study, the short-matrix EPDS and the AAS were administered through a tablet questionnaire, and based on self-reported retrospective information. Thus, the answers may be influenced by recall bias. The low rate of participants in the Safe Pregnancy study (33.5%) causes concerns as it might have introduced selection bias and affected the generalizability of findings. Women in our study share many characteristics associated with lower risk of experiencing antenatal depression and IPV (older, employed, married, high education level)^1,9^. Further, more women with a non-native Norwegian background declined to participate in the study^34^. The results in our study might be generalized to women with similar socioeconomic and sociocultural backgrounds attending antenatal care in Norway and in other countries. Due to the low number of participants from our target populations, the study results are not generalizable to women with Pakistani and Somali cultural backgrounds.

Some women may have felt uncomfortable reporting sensitive information about antenatal depression and experiences of IPV. This may have led to underreporting and thus consequently an underestimate of the prevalence of antenatal depression and IPV as well as that a weaker association exists. Measures were taken to facilitate a safe and supportive environment for disclosure of symptoms of antenatal depression and IPV. Women were only recruited when they met with the midwife alone, they were given privacy to answer the tablet questionnaire, informed multiple times that their answers were anonymous and encouraged to speak with the midwife if they wanted to after completing the questionnaire^22^.

Translation of the consent form and the questionnaire as well as a qualitative user-involvement study^30^ was conducted to facilitate recruitment of women with Pakistani and Somali backgrounds. Despite this, it was not enough to recruit more women from our target groups. Non-native Norwegian speaking women in our study may have originated from high-, middle-, and low-income countries. Nevertheless, they may have a cultural and linguistic barrier when communicating about sensitive topics^35^.

## CONCLUSIONS

Our findings suggest that symptoms of depression are prevalent among pregnant women. The prevalence of symptoms of antenatal depression did not differ significantly for native and non-native Norwegian speakers. The women who report symptoms of antenatal depression were more likely to have experienced physical, emotional and/or sexual IPV in their lifetime compared to women with no symptoms of depression. More knowledge and awareness about the association between symptoms of depression in the perinatal period and IPV across different ethnic, cultural, socioeconomic and sociodemographic groups are warranted. Studies need to attempt to recruit women with immigrant backgrounds as they are likely to be at high risk of experiencing symptoms of antenatal depression but also are likely to be missed by health services. To work for a better identification of women at risk that meet the specific needs of an increasingly heterogeneous populations is needed.

## Data Availability

The data supporting this research are available from the authors on reasonable request.
